# Urban and Rural Disparities of Personal Health Behaviors and the Influencing Factors During the COVID-19 Outbreak in China: Based on an Extended IMB Model

**DOI:** 10.1017/dmp.2020.457

**Published:** 2020-11-19

**Authors:** Yetao Luo, Lili Yao, Ling Hu, Li Zhou, Feng Yuan, Xiaoni Zhong

**Affiliations:** 1 Department of Nosocomial Infection Control, Second affiliated Hospital, Army Medical University, Chongqing, China; 2 Department of Anesthesiology, the First Affiliated Hospital of Chongqing Medical University, Chongqing, China; 3 Department of Epidemiology and Health Statistics, School of Public Health and Management, Chongqing Medical University, Chongqing, China; 4 Department of Gastroenterology, The Second Affiliated Hospital of Chongqing Medical University, Chongqing, China; 5 Intensive Care Unit, Children’s Hospital Affiliated to Chongqing Medical University, Chongqing, China

**Keywords:** COVID-19, health behaviors, information motivation behavior skills model, perceived stress

## Abstract

**Objective:**

Health behavior was conducive to control the coronavirus disease (COVID-19) epidemic. This study aimed to determine the differences in health behaviors and related factors among rural and urban residents in China.

**Methods:**

From February 14 to 22, 2020, during the peak of the COVID-19 epidemic in China, a total of 2449 participants (1783 (72.81%) urban residents and 666 (27.19%) rural residents) were recruited by snowball sampling on WeChat and QQ social platforms, both owned by Tencent. Data were collected through the Web-questionnaire guided by an information–motivation–behavioral skills model. The multiple-group structural equation model was applied to analyze the factors.

**Results:**

Rural residents had lower health behavior scores than urban residents, even after adjusting demographic characteristics (33.86 vs 34.29, *P* = 0.042; total score was 40). Motivational, behavioral skills, and stress had direct positive and negative influences on health behaviors of urban and rural residents. Information and positive perception of interventions had direct effects on health behaviors in rural residents, but not in urban residents. All the factors were mediated by behavioral skills in rural and urban residents.

**Conclusions:**

This study suggests that the government should pay attention to substantial rural and urban disparities and implement different COVID-19 prevention and intervention policies for health behaviors targeting rural and urban residents.

The 2019 coronavirus disease (COVID-19) caused by severe acute respiratory syndrome coronavirus 2 (SARS-CoV-2) has posed a serious threat to the public health and economic and social development globally. The World Health Organization has raised the risk assessment of this disease as very high. As of May 13, 2020, 4 170 424 confirmed cases and 287 399 deaths were reported worldwide.^1^

In addition to policies that were implemented by the Chinese Government and other relevant departments, residents’ health behaviors, such as following management, taking corrective measures to wear masks, reducing outings, and maintaining a positive mentality, were also crucial for reducing the risk of infection and controlling the epidemic.

Even though the urban–rural gap in China is gradually decreasing, in comparison with urban areas, families who live in rural regions have lower levels of education, socioeconomic status, and medical resources.^2,3^ Therefore, it can be expected that the health behaviors and their influencing factors may vary between urban and rural residents. It also is a very important consideration in determining comprehensive intervention strategies applicable to rural and urban areas.

Therefore, based on the extended information–motivation–behavioral (IMB) skills model that included information, motivation, behavioral skills, perceived stress, positive perception of interventions and health behaviors and can be easily converted into intervention practice, this study accessed urban–rural disparities on the influencing factors of health behaviors during the COVID-19 outbreak in China, so as to give insight for targeted intervention measures and policies for other countries and regions.

## Materials and Methods

### Study Design and Participants

This cross-sectional online survey was conducted from February 14 to 22, 2020, the peak of the COVID-19 epidemic in China. Participants were recruited by snowball sampling on Tencent’s WeChat and QQ social platforms, using the online survey tool of questionnaire star to make structured questionnaires guided by the IMB model, and collecting data through the Web-questionnaire method in China. Each participant can become a seed and expand the sample by sharing the questionnaire to his or her social network. Participants had to read the informed consent and agree to participate before filling out the formal questionnaire. Inclusion criteria were (1) age ≥ 18 years old; (2) ability to use smart electronic devices (eg, computers, tablets, mobile phones); and (3) agreeing to participate in the study. Exclusion criteria were (1) suffering from psychological diseases such as anxiety and depression and (2) response time of less than 1 minute or more than 30 minutes.

### Quality Control

In order to reduce bias, quality control had been carried out: (1) Each item in the questionnaire was set as a required question to ensure the integrity of the data; (2) each IP could be submitted only once to avoid repeated filling; and (3) real-time monitoring in the background and response time of participants were recorded.

### Measures

The description of the questionnaire is shown in the Supplementary Table 1. The higher the score in each construct, the higher the information, motivation, behavioral skills, positive perception of the government, the perceived stress, and the frequency of health behaviors during the COVID-19 epidemic. Especially, the correct answer of information would appear after submitting the questionnaires. Positive perception of interventions refers to residents’ perceptual evaluation of the government’s epidemic prevention and control measures, and the contents were the understanding, support, and evaluation of the interventions. The Chinese version of the Perceived Stress Scale was used in this study, with a total of 14 items, and the total scores were higher than 25, representing *health risk stress*.^4,5^

### Statistical Analysis

Normal distribution variables were reported by mean and standard error (SE). The generalized linear regression model was used to test the differences between the urban and rural residents after adjusting for age, gender, education, marital status, personal monthly income, and occupation. Categorical variables were reported as numbers (n) and percent (%), and the chi-squared test was used to test the difference between the 2 groups. The confirmatory factor analysis (CFA) was used to test the relationship between latent variables and observable variables of the measurement model. Multiple-group structural equation modeling (MSEM) was used to test whether the relationship among constructs in the extended model was invariant between rural residents and urban residents in Amos 24.0. Statistical analysis was performed using SAS 9.4 (SAS Institute, Inc., Cary, NC). A *P*-value < 0.05 was considered significant.

## Results

### Participant Characteristics

This survey included 2449 valid participants (n = 1783 [72.81%] urban residents and n = 666 [27.19%] rural residents) after excluding 104 participants. Table 1 presents the sociodemographic characteristics and information of rural and urban participants in this study. There was significant difference in age, gender, education level, marital status, personal monthly income, province of residence, and occupation between the urban and rural residents (all *P* < 0.05; see Table 1). Rural residents wore masks and reduced group gathering activities less frequently than urban residents (both *P* < 0.05; see Table 1). Urban residents had a significantly higher level of information, motivation, behavioral skills, and health behaviors compared with rural residents after adjusted confounding factors (all *P* < 0.05; see Table 1). There were no significant rural–urban differences for perceived stress and positive perception of intervention (both *P* > 0.05; see Table 1). Rural residents had a lower correct percent in knowledge questions I1 to I5 and I7 than urban residents (all *P* < 0.05; see Table 1).

**Table 1.** Participant characteristics (n = 2449)

Variables	Total	Urban	Rural	*P*-value
N	2449	1783	666	
***Sociodemographic Characteristics, N (%)***				
**Gender**				
Male	823 (33.61)	550 (30.85)	273 (40.99)	< 0.001
Female	1626 (66.39)	1233 (69.15)	393 (59.01)	
**Age, years**				
18-30	1300 (53.08)	1010 (56.65)	290 (43.54)	< 0.001
∼ 50	913 (37.28)	617 (34.60)	296 (44.44)	
≥ 51	236 (9.64)	156 (8.75)	80 (12.01)	
**Education level**				
Junior high school or below	374 (15.27)	169 (9.48)	205 (30.78)	< 0.001
Senior high school diploma (or) advanced diploma	1038 (42.38)	779 (43.69)	259 (38.89)	
Baccalaureate degree or above	1037 (42.34)	835 (46.83)	202 (30.33)	
**Marital status**				
Non-married	1000 (40.83)	789 (44.25)	211 (31.68)	< 0.001
Married	1353 (55.25)	927 (51.99)	426 (63.96)	
Divorced or widowed	96 (3.92)	67 (3.76)	29 (4.35)	
**Personal monthly income, RMB**				
≤ 3000	845 (34.50)	482 (27.03)	363 (54.50)	< 0.001
∼ 5000	880 (35.93)	663 (37.18)	217 (32.58)	
≥ 5001	724 (29.56)	638 (35.78)	86 (12.91)	
**Province of residence**				
Chongqing	1408 (57.49)	1144 (64.16)	264 (39.64)	< 0.001
Sichuang	434 (17.72)	271 (15.20)	163 (24.47)	
Others	607 (24.79)	368 (20.64)	239 (35.89)	
**Occupation**				
Job-holders	1834 (74.89)	1352 (75.83)	482 (72.37)	< 0.001
Students	261 (10.66)	206 (11.55)	55 (8.26)	
Jobless or job-waiting individuals	268 (10.94)	175 (9.81)	93 (13.96)	
Retirees	86 (3.51)	50 (2.80)	36 (5.41)	
***Health Behaviors, N (%)***				
**Reduce group gathering activities such as going out and gathering**				
Never to sometimes	231 (9.43)	172 (9.65)	59 (8.86)	0.014
Often	951 (38.83)	661 (37.07)	290 (43.54)	
Always	1267 (51.74)	950 (53.28)	317 (47.60)	
**Wearing a mask when going out**				
Never to sometimes	127 (5.19)	79 (4.43)	48 (7.21)	< 0.001
Often	313 (12.78)	176 (9.87)	137 (20.57)	
Always	2009 (82.03)	1528 (85.70)	481 (72.22)	
***Score, Mean±SE*** ^a^				
Information	4.34±0.03	4.14±0.09	4.03±0.10	0.019^a^
Motivation	19.45±0.06	19.22±0.19	18.88±0.22	0.009^a^
Behavioral skills	32.14±0.06	32.32±0.20	31.42±0.22	< 0.001^a^
Health behaviors	34.62±0.09	34.29±0.32	33.86±0.36	0.042^a^
Positive perception of interventions	22.25±0.06	22.23±0.21	22.07±0.24	0.283^a^
Perceived stress	22.25±0.15	21.97±0.51	22.09±0.58	0.727^a^
***Information, N (%)***				
I1: Antibiotics could not prevent COVID-19	1912 (78.07)	1431 (80.26)	481 (72.22)	< 0.001
I2: Taking shuanghuanglian oral liquid could not prevent COVID-19	1982 (80.93)	1473 (82.61)	509 (76.43)	0.001
I3: Room fumigated vinegar could not kill SARS-CoV-2	1977 (80.73)	1474 (82.67)	503 (75.53)	< 0.001
I4: Wear gauze masks or activated carbon masks correctly to prevent COVID-19	2002 (81.75)	1509 (84.63)	493 (74.02)	< 0.001
I5: Hot water at 56°C for 30 minutes could kill SARS-CoV-2	1819 (74.28)	1377 (77.23)	442 (66.37)	< 0.001
I6: In general, the longest incubation period for COVID-19 is 14 days	2251 (91.92)	1649 (92.48)	602 (90.39)	0.091
I7: The main transmission method of COVID-19 is droplet transmission and contact transmission	2147 (87.67)	1593 (89.34)	554 (83.18)	< 0.001

*Notes*:

^a^Adjust gender, age, education level, marital status, personal monthly income, province of residence and occupation by generalized linear regression model.

SE = standard error.

### MSEM Analysis

**Figure 1.**
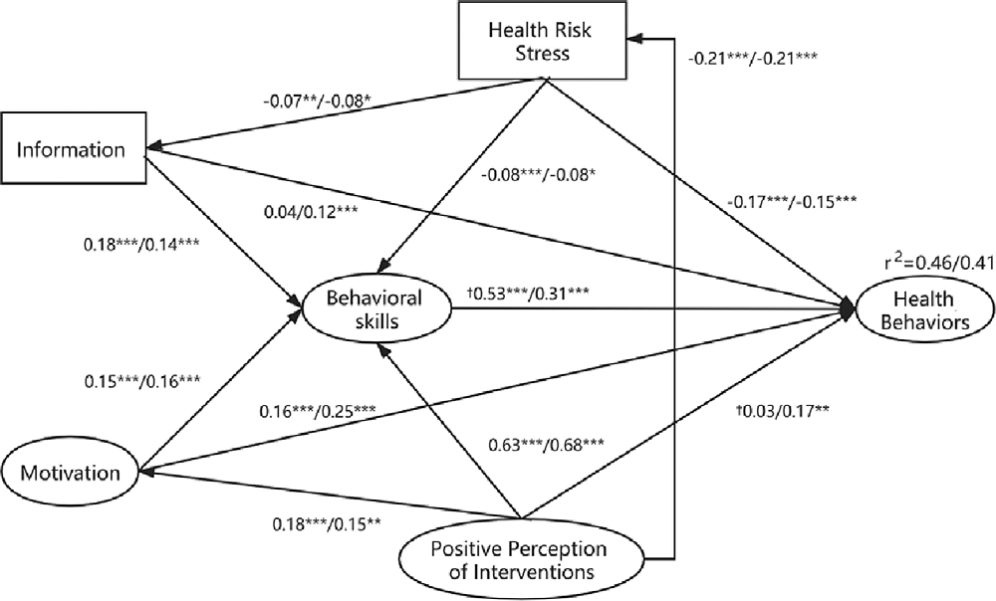
Multigroup comparison of the final extended IMB model applied to urban and rural area (urban residents, N = 1783; rural residents, N = 666). *P < 0.05; **P < 0.01; ***P < 0.001. ^†^Difference of standardized path coefficients is statistically significant between urban residents and rural residents.

All measurement model had a good fit with incremental fit index (IFI) > 0.9, confirmatory fit index (CFI) > 0.9, and root mean square error of approximation (RMSEA) < 0.08 (Supplementary Table 1). Model fit indices results, χ^2^/df ratio = 1865.966/642 = 2.907, goodness-of-fit index (GFI) = 0.940, CFI = 0.937, IFI = 0.931, RMSEA = 0.029, indicated that the final model had a good fit with the data for both groups (Figure 1). All path coefficients were statistically significant (all *P* < 0.05).

Figure 1 shows the path coefficients of latent variables. In urban residents and rural residents, 3 factors had significant direct effects on health behaviors, namely, motivation, behavioral skills, and health risk stress, and the first also exerted indirect impacts on health behaviors through behavioral skills (all *P* < 0.05). Health risk stress had negative, indirect effects on health behaviors through information and behavioral skills, and positive perception of interventions had positive, indirect effects through motivation, behavioral skills, and health risk stress in all residents (all *P* < 0.05).

In rural residents, information and positive perception of interventions had significant direct and indirect effects on health behaviors, but, in urban residents, only indirect effects. Compared with the control group, rural residents had significantly higher path coefficients in “Positive Perception of Intervention → Health Behaviors” (0.17 vs 0.03, t = 2.14, *P* < 0.05), but significantly smaller path coefficients in “Behavioral Skills → Health Behaviors” (0.31 vs 0.53, t = 2.87, *P* < 0.05). The total effect coefficients of information, motivation, behavioral skills, health risk stress, and positive perception of interventions on health behaviors were 0.14, 0.25, 0.53, -0.22, and 0.45 in urban residents, respectively, and these coefficients were 0.17, 0.31, 0.30, -0.19, and 0.47, respectively, in rural residents. Finally, the extended IMB model accounted for 46% of health behaviors for urban residents and 41% for rural residents (see Figure 1).

## Discussion

Participants in this study had a high level of health behaviors during the COVID-19 epidemic (the percentages of always wearing a mask when going out and often reducing group gathering activities were 82.03% and 90.67%, respectively). This study reveals that rural residents had lower health behavior scores than urban residents, even after adjusting demographic characteristics. There are differences in the factors affecting the health behaviors of urban and rural residents. First, information has direct effects on rural health behaviors, which means information is good for prevention, but not among urban residents. Information or knowledge about disease transmission and self-protection behavior is identified as one of the determinants of behavioral changes in most behavioral intervention models. However, this study indicated that residents had limited knowledge about COVID-19, especially residents in rural areas. The false knowledge may lead to ineffective preventive measures taken by residents and increase the risk of infection.

Second, the path coefficient of behavioral skills to health behaviors among urban residents was higher than that among rural residents (0.53 vs 0.31). This seems to imply that behavioral skills affect urban residents more deeply than rural residents. Behavioral skills are indispensable for improving health behaviors,^6^ and long-term prevention behaviors also depend on behavioral skills.^7^ Third, positive perception of interventions has a direct impact on health behavior in rural residents, but not in urban residents. This shows that, although urban and rural positive perceptions of intervention scores were similar, their effects on health behaviors were different.

Health risk stress had negative effects on health behaviors in urban and rural residents. Excessive stress may lead to negative coping styles, and only those who maintain a high level of awareness of danger and maintain a moderate level of stress are most likely to adopt appropriate health behaviors.^8^ Furthermore, positive perception of interventions had negative effects on health risk stress. This revealed that the prevention and control measures adopted by the Chinese Government and relevant organizations can reduce the fear and anxiety of residents and enhance health behaviors.

This study had limitations: (1) The randomness of the samples was poor. We used a large sample to ensure that there was a certain number of individuals in all categories, to minimize bias; (2) results extrapolation was limited, to some extent. It mainly represented the regions of Chongqing and Sichuan or regions with similar epidemic severity. In view of the international nature of COVID-19 and its implications, future studies should include a broader sample.

## Conclusion

In general, information, motivation, behavioral skills, health risk stress, and positive perception of interventions were good explanatory variables of health behaviors, but their paths and coefficients on health behaviors were not consistent between rural and urban residents. This study provides possible evidence to support the need to implement different COVID-19 prevention and intervention policies for health behaviors targeting rural and urban residents.

## Supplementary material

For supplementary material accompanying this paper visit https://doi.org/10.1017/dmp.2020.457. click here to view supplementary material
