# Supplementary material for: Urban and Rural Disparities of Personal Health Behaviors and the Influencing Factors During the COVID-19 Outbreak in China: Based on an Extended IMB Model
Source: Disaster Med Public Health Prep. 2020 Nov 19:1–5. doi: 10.1017/dmp.2020.457 (PMC7985648; doi:10.1017/dmp.2020.457)
Supplement: Supplementary file 1 [file S1935789320004577sup001.doc]

| Supplementary table 1.Constructs for Information, Motivation, Behavioral Skills, Healthy Behaviors and Positive Perception of Interventions. | | | | | | | |
| --- | --- | --- | --- | --- | --- | --- | --- |
| Constructs items( score range) | | May answer | β | p-Value | IFI/CFI | RMSEA | α |
| **Perceived Stress(0-56)** | | A five-point Likert scale, ranging from 1 (never) to 5 (always) |  |  |  |  |  |
|  | Score |  |  |  |  |  | 0.829 |
| **Information(0-7)** | | "Yes", "No" and "Don't know" |  |  |  |  |  |
|  | Knowledge score |  |  |  |  |  | 0.607 |
| I1 | Could antibiotics prevent COVID-19? |  |  |  |  |  |  |
| I2 | Could taking shuanghuanglian oral liquid prevent COVID-19? |  |  |  |  |  |  |
| I3 | Could room fumigated vinegar kill SARS-CoV-2? |  |  |  |  |  |  |
| I4 | Could gauze masks or activated carbon masks prevent COVID-19? |  |  |  |  |  |  |
| I5 | Could hot water at 56 degrees celsius kill SARS-CoV-2 for 30 minutes? |  |  |  |  |  |  |
| I6 | In general, is the longest incubation period for COVID-19 14 days? |  |  |  |  |  |  |
| I7 | Is COVID-19 the main transmission method by droplet transmission and contact transmission? |  |  |  |  |  |  |
| **Motivation(5-25)** | | A five-point Likert scale, ranging from 1 (completely disagree) to 5 (completely agree) |  |  |  |  |  |
| M1 | I thought the COVID-19 was very contagious |  | 0.470 | <0.001 | 0.973/0.972 | 0.054 | 0.639 |
| M2 | Taking health behaviors could reduce the risk of infection. |  | 0.413 | <0.001 |  |  |  |
| M3 | I was very afraid of COVID-19 |  | 0.510 | <0.001 |  |  |  |
| M4 | I thought COVID-19 was serious |  | 0.669 | <0.001 |  |  |  |
| M5 | My family was very supportive of my health behaviors |  | 0.476 | <0.001 |  |  |  |
| **Behavioral Skills(7-35)** | | A five-point Likert scale, ranging from 1 (completely disagree) to 5 (completely agree) |  |  |  |  |  |
| BS1 | I actively paid attention to Real-time Information of COVID-19 |  | 0.415 | <0.001 | 0.961/0.960 | 0.060 | 0.768 |
| BS2 | I understood the importance of home isolation during COVID-19 epidemic |  | 0.451 | <0.001 |  |  |  |
| BS3 | I grasped the difference between a common cold and COVID-19 |  | 0.444 | <0.001 |  |  |  |
| BS4 | I could put on the mask correctly |  | 0.671 | <0.001 |  |  |  |
| BS5 | I could take temperature correctly |  | 0.675 | <0.001 |  |  |  |
| BS6 | I could follow community or village committee regulations |  | 0.630 | <0.001 |  |  |  |
| BS7 | I took the initiative to learn preventive measures for COVID-19 |  | 0.609 | <0.001 |  |  |  |
| **Positive Perception of Interventions(5-25)** | | A five-point Likert scale, ranging from 1 (completely disagree) to 5 (completely agree). |  |  |  |  |  |
| PPI1 | Prevention and control measures increased my confidence |  | 0.784 | <0.001 | 0.968/0.968 | 0.043 | 0.890 |
| PPI2 | I understanded the prevention and control management measures |  | 0.800 | <0.001 |  |  |  |
| PPI3 | I supported prevention and control management measures such as closed management |  | 0.785 | <0.001 |  |  |  |
| PPI4 | Prevention and control measures could reduce my fear and anxiety |  | 0.750 | <0.001 |  |  |  |
| PPI5 | Prevention and control measures could prevent me and my family from contracting the COVID-19. |  | 0.758 | <0.001 |  |  |  |
| **Health Behaviors(8-40)** | | A five-point Likert scale, 1 (never) to 5 (always) |  |  |  |  |  |
| HB1 | Advise family members to wash their hands frequently, wear masks, etc. |  | 0.606 | <0.001 | 0.983/0.983 | 0.049 | 0.844 |
| HB2 | Wash hands frequently at home |  | 0.760 | <0.001 |  |  |  |
| HB3 | Reduce group gathering activities such as going out and gathering. |  | 0.711 | <0.001 |  |  |  |
| HB4 | Keep a safe distance from strangers when going out (at least 1 meter) |  | 0.718 | <0.001 |  |  |  |
| HB5 | Cover mouth and nose with a tissue or elbow when coughing or sneezing to avoid others |  | 0.707 | <0.001 |  |  |  |
| HB6 | Wearing a mask when going out |  | 0.561 | <0.001 |  |  |  |
| HB7 | Health diet to improve nutrition level |  | 0.584 | <0.001 |  |  |  |
| HB8 | Take appropriate exercises at home |  | 0.430 | <0.001 |  |  |  |
| IFI/CFI, incremental fit index/comparative fit index; RMSEA, root mean square error of approximation. | | | | | | | |
